# Can transcranial direct current stimulation aimed at reducing mind wandering alleviate depression?

**DOI:** 10.3389/fpsyt.2025.1653616

**Published:** 2025-09-24

**Authors:** Juergen Fell

**Affiliations:** Department of Epileptology, University Hospital Bonn, Bonn, Germany

**Keywords:** mind wandering, depression, tDCS, DLPFC, rIPL

The investigation of mind wandering (MW) has become a prominent research topic, not only in cognitive neuroscience, but now also in clinical neuroscience. For the purpose of this article, MW will be understood to be a state in which attention is withdrawn from the task or situation at hand and drifts to internal thoughts, imaginings or feelings (1). While the positive effects of MW, such as enhanced autobiographical planning, creativity, and problem solving, are a key focus in cognitive neuroscience (2), its negative effects are of particular relevance to clinical neuroscience. Notably, studies have shown a bidirectional relationship between excessive MW, i.e.MWoccurring with a significantly higher-than-typical frequency, and negative affect (3–5). Another factor closely linked to MW and affect is sleep quality. These three elements, excessive MW, negative effect, and poor sleep, have been proposed to form a self-reinforcing vicious cycle (6). Consequently, excessive MW has been suggested as a contributing factor to the development of psychiatric disorders, particularly major depression (7, 8). The intervention proposed below targets excessive MW.

It is well-know that rumination, a specific type of MW characterized by its repetitive nature, negative, and self-related content, as well as thematic uniformity, is a major characteristic of patients suffering from major depression. Rumination in depression has been extensively studied and has been linked to both the onset (9) and maintenance of the disorder (10), as well as to its severity, including an increased risk of suicidality (11). However, few studies have examined how overall MW, beyond rumination, relates to depression (for an overview, see 8). Most of these studies have relied on self-report questionnaires, which heavily depend on participants’ self-awareness and fidelity. A more reliable method is experience sampling, which involves probing individuals about their current attentional state at random intervals over a given time period or during an attentional task. Nevertheless, studies using experience sampling in depression have had several limitations, including small sample sizes, exclusive focus on specific tasks, short sampling periods, and imprecise categorization of MW and rumination. Taken together, the findings have been inconclusive regarding whether overall MW is increased in major depression or not (8).

Recently, Welhaf et al. (12) addressed this question in a well-designed and methodologically rigorous study. They investigated 53 patients with major depression and 53 matched healthy controls (average BDI-II scores: 32.2 versus 1.7). Using a hand-held electronic device, participants’ experiences were sampled 8 times per day over the course of one week. The experience sampling probes assessed MW, its emotional valence, temporal orientation, positive and negative affect (11 items), and brooding (5 items; ruminative response scale, 13). The authors found that overall MW was significantly increased - and more than twice as frequent - in depression patients compared to controls (average MW propensities: 0.37 versus 0.17; p < 0.001). In other words, their findings indicate the presence of excessive MW in individuals with depression. As expected, MW in depressed individuals was more negatively and less positively valenced. Moreover, in patients - but not in controls - increases in MW were significantly associated with higher negative affect and lower positive affect, even when controlling for rumination. Based on these findings, is appears evident that reducing the amount of overall MW may help alleviate symptoms of major depression.

A well-known and relatively simple non-invasive brain stimulation approach for influencing MW is transcranial direct current stimulation (tDCS). tDCS has been shown to increase or decrease the excitability of cortical areas, depending on whether anodal or cathodal stimulation is applied (14). Several investigations have explored whether MW can be modulated through tDCS (for an overview, see 15). These studies targeted cortical regions either associated with MW (16), such as default mode network (DMN) areas including the medial prefrontal cortex (mPFC), or regions thought to support attentional control and task-focus, such as the dorsolateral prefrontal cortex (DLPFC). However, the study protocols varied considerably, and the outcomes were highly inconsistent.

In light of this, Nawani et al. (17) conducted a meta-analysis of 15 sham-controlled tDCS studies on MW to evaluate whether there is overall evidence for an effective target site. They assessed the risk of bias based on 11 criteria, extracted effect sizes (real versus sham stimulation) using Cohen’s d, and performed electric field modelling based on a standard head model (Montreal Neurological Institute). As the central focus of the analysis, electric fields perpendicular to the cortical surface were calculated for two regions of interest: the left dorsolateral prefrontal cortex (lDLPFC) and the right inferior parietal lobule (rIPL). A random effects meta-analysis, incorporating 11 different models with effect size as the outcome variable, suggested that tDCS targeting the lDLPFC did not significantly influence MW, as reflected in the effect sizes. In contrast, the analysis indicated that anodal stimulation of the rIPL was a significant predictor of reductions in MW, even after controlling for risk of bias. The rIPL is a prominent region implicated in MW, as evidenced in a meta-analysis of neuroimaging studies (16). It is considered a key component of the DMN (18), while also playing a central role in multisensory integration and stimulus-driven attention as a part of the frontoparietal control network (19). This dual involvement suggests that the rIPL is well-positioned to mediate shifts between external and internal focus. Based on this notion, along with the outcome of the meta-analysis by Nawani et al. (17), and the observation of markedly increased MW in major depression by Welhaf et al. (12): could the rIPL represent an effective target region for tDCS interventions aimed at alleviating depression symptoms?

The use of tDCS for the treatment of major depression has been extensively investigated in numerous studies. While existing meta-analyses generally agree that tDCS, compared to sham stimulation, is effective in reducing depression symptoms (20–23), some have also reported superior response and remission rates (21, 22), whereas others did not find significant evidence for such effects (20, 23). The vast majority of tDCS studies in depression have targeted the DLPFC, mostly using anodal stimulation of the left DLPFC. Owing to its role in attentional control, cognitive flexibility, and mood regulation, this region is considered a promising tDCS target for several psychiatric disorders, including depression, schizophrenia, attention deficit hyperactivity disorder, and substance use disorders (24). A meta-analysis of tDCS studies aimed at reducing depressive rumination identified nine studies, all of which used the DLPFC as the target region (25). Of these, five reported a significant impact of tDCS on rumination, while four did not. Apart from the DLPFC, only a few studies have investigated alternative target regions in depression, such as the ventrolateral PFC (26) - part of the executive control network, or the dorsomedial PFC (27) - a DMN region.

Is there evidence that tDCS targeting the rIPL may be effective in alleviating depression symptoms, beyond its potential as a target for modulating MW? A search of Pubmed and Google (keywords: tDCS, parietal, depression) identified only one study to date that has used this target region in the context of depression. Guo et al. (28) applied anodal tDCS over the right parietal cortex in 12 patients with major depression. The study was based on the idea that the right parietal cortex is involved in attention to and monitoring of exogenous information, and that anodal stimulation may enhance this function, thereby reducing an excessive focus on endogenous information. Participants were either medication-free for at least one month prior to enrolment or were taking antidepressants with stable dosing but poor treatment response. Stimulation was delivered with a 35 cm^2^ electrode positioned at P4 (10-20 system), which is located near the rIPL. TDCS was administered for 20 min, twice daily, over a two-week period. The authors reported a significant reduction in depression symptoms, with average Hamilton Depression Rating Scale-17 scores decreasing from 23.4 at baseline to 9.0 post-treatment. However, due to the small sample size and absence of a control group, this study provides only tentative evidence for the efficacy of rIPL-targeted tDCS in depression. Moreover, it is important to note that MW was not assessed in this study. Thus, the rationale outlined above is not directly supported by these results. Further research with larger samples, ideally using randomized, sham controlled designs and incorporating measures of both depression and MW, is needed to confirm this initial finding.

In summary, excessive MW may contribute to the origin and development of depression (8). Recent findings indicate that overall MW is more than twice as prevalent in individuals with major depression (12). A meta-analysis (17) suggests that anodal tDCS targeting the rIPL can reduce MW, consistent with the rIPL’s role in both attentional networks and the DMN (18, 19). These results imply that tDCS targeting the rIPL may be effective in mitigating excessive MW in depression. Although tDCS has been extensively studied in depression, its application using the rIPL as the target region remains largely under investigated. Therefore, randomized, sham-controlled tDCS studies targeting the rIPL in depression are urgently needed.
